# Editorial: Resilience of mental health professionals following the COVID-19 pandemic

**DOI:** 10.3389/fpsyt.2023.1227848

**Published:** 2023-06-20

**Authors:** Anneloes van den Broek, Matyas Galffy, Lars de Vroege

**Affiliations:** ^1^Tranzo Department, Tilburg School of Social and Behavioral Sciences, Tilburg University, Tilburg, Netherlands; ^2^Department of Anxiety and Depression, GGz Breburg, Breda, Netherlands; ^3^Breburg Academy, Department of Post Academic Psychology Training and Education, GGz Breburg, Tilburg, Netherlands; ^4^University Clinic for Psychiatry II, Department of Psychiatry, Psychotherapy and Psychosomatics, Innsbruck Medical University, Innsbruck, Austria; ^5^Clinical Centre of Excellence for Body, Mind, and Health, GGz Breburg, Tilburg, Netherlands

**Keywords:** mental health professionals, COVID-19, waiting list, postponed care, resilience, training

Nowadays, the effects of the COVID-19 pandemic are getting clearer in mental health care because patients finished their physical rehabilitation programs within somatic health care. As a result, more and more patients enroll in mental health care institutions with (long-lasting) mental health complaints. This leads to a higher workload for healthcare professionals as well as longer waiting lists for patients.

The current Research Topic aims to explore the long-term consequences of the pandemic on mental health professionals. Moreover, as a large amount of professionals (the long standing ones) experience these consequences differently, we are interested in exploring how their resilience enables them to remain mentally healthy. This difference in coping behavior of mental health professionals would be, in turn, interesting for developing future training policy. This Research Topic aims to collect manuscripts on mental health of professionals working in (but not limited to) mental health institutions, the role of resilience and other personality traits that are linked with better mental health, and (both short-term and long-term) support programs to support mental health professionals with regards to their mental health status as a result of the COVID-19 pandemic.

This series consists of eight articles of different study designs addressing the following questions: What is the role of resilience? What kind of personality characteristics are associated with mental health? Furthermore, how can mental health and resilience be promoted amongst mental health professionals?

Chen et al. reported that medical staff, who worked during the pandemic, burnout was affected mostly by anxiety and depression. Resilience of employees, especially psychological resilience, had a mediation role in this effect and therefore the authors concluded that monitoring (negative) emotional state of medical staff is pivotal in order to improve (psychological) resilience and decrease burnout. The results of Chen et al. contribute to the body of evidence that monitoring mental state of medical staff is necessary in order to prevent burnout development. Furthermore, in order to increase (psychological) resilience, such monitoring is necessary in order to prevent decreasing mental state timely and improve or support resilience on time.

Besides burnout, the risk of developing post-traumatic stress disorder (PTSD) and somatization among healthcare workers is high. Perceived stress amongst frontline healthcare workers fighting against COVID-19 is high and Li et al. concluded that the prevalence of PTSD and somatic symptoms amongst mental health of frontline healthcare workers deserves more attention. Their results show that resilience was negatively associated with PTSD and somatization, and the relationship among resilience, PTSD, and somatization was mediated by perceived stress. Therefore, their results further show that resilience and maintaining resilient is key in order to improve mental state and (try to) prevent development of, e.g., PTSD.

Even though more and more studies commit to the topic of this Research Topic, still little is known about the mental health of specific psychotherapists during the COVID-19 pandemic. Schaffler et al. assessed mental health during the COVID-19 pandemic amongst Austrian psychotherapists and compared these findings with the prevalence of the general population. Psychotherapists showed lower odds for exceeding cut-offs for clinically relevant depressive, anxiety, insomnia and stress symptoms compared to the general population (Schaffler et al.) which may indicate that psychotherapists are more resilient but the current data prevents, sadly, the interpretation of any causal relationships.

Failla et al. conducted an online study in European (France, Italy, Portugal, and Spain) public health residents during the COVID-19 pandemic. Their results indicated that the pandemic had a significant impact on 43 public health residents in terms of depression, anxiety, and stress, especially for women and who lost work-related opportunities (Failla et al.).

During the COVID-19 pandemic, the Chinese government adopted a centralized isolation treatment (CIT) strategy for patients in mobile cabin hospitals. Although this greatly improved the efficiency of the pandemic response, the medical employees in mobile cabin hospitals, suffered more from mental health complaints compared to those in local hospitals. The study of Song et al. explored how the CIT strategy had impact on the medical staff's mental health. Their results showed that mental health of anti-COVID-19 medical staff in mobile cabin hospitals adopting CIT was worse compared to local hospitals and the mental health recovery of medical staff in CIT hospitals was slower than in local hospitals (Song et al.). These results are important for the ongoing implementation of the program. After all, stability of mental health of healthcare workers is necessary to continue the CIT-strategy.

A scoping review of our own group explored whether there is a moderating effect of the COVID-19 pandemic on the mental wellbeing of Healthcare workers in the context of sustainable employability. van den Broek et al. concluded that mental problems amongst health care workers were already abundantly present before the COVID-19 pandemic. What changed during the pandemic was that mental health problems have increased in prevalence, severity, and variation. In general, a negative relation between (mental) health and sustainable employability exists. The literature review showed that mental health problems impacted sustainable employability of health care workers heavily: absenteeism has increased and perspective on work has changed (van den Broek et al.).

To explore the potential mediating role of resilience in the relationship between perceived social support and the sense of security of Chinese medical personnel, a multi-stage proportionally stratified convenience sampling method was adopted to select 4,076 medical professionals from 29 hospitals in Guangdong Province by He et al.. They concluded that perceived social support and resilience were positively associated with a sense of security and perceived social support was positively associated with resilience. Structural equation modeling revealed that resilience played a partial mediating role in the association between perceived social support and a sense of security (He et al.).

Lastly, Willmund et al. estimated pandemic related impacts using an anonymous personnel survey which was conducted in two German military hospitals compared to the hospital staff in general vs. the psychiatric personnel at two measurement time points. The results suggest good adaptative skills within the psychiatric staff in military hospitals, which could be interpreted as a sign for good resilience (Willmund et al.). This might have led to lower stress related symptoms during the COVID-19 pandemic.

We hope that our series have contributed to better understanding of resilience and mental health amongst health care workers during the COVID pandemic. The impact of the pandemic is, however, still lasting and affecting daily-life; long COVID consequences are getting clearer, the persistent mental health care symptoms are more apparent, and the need for support of (mental) health care workers remains pivotal in the future.

## Author contributions

All authors listed have made a substantial, direct, and intellectual contribution to the work and approved it for publication.

